# Bulk and single-cell multiomics insights into theca cell and granulosa cell dysfunction in polyendocrine metabolic ovarian syndrome

**DOI:** 10.1186/s13048-026-02226-3

**Published:** 2026-08-07

**Authors:** Supraja M. Kodanch, Vaishnavi Vyas, Saumya Bansal, Biligerepalya S. Chinmayi, Shashikala K. Bhat, Prashanth K. Adiga, Shama Prasada Kabekkodu, Padmalatha S. Rai

**Affiliations:** 1 Department of Biotechnology, Manipal School of Life Sciences, Manipal Academy of Higher Education, Manipal, Karnataka 576104 India; 2 Department of Obstetrics and Gynaecology, Dr T M A Pai Hospital, Udupi, Karnataka 576101 India; 3 Department of Reproductive Medicine and Surgery, Kasturba Medical College, Manipal, Karnataka 576104 India; 4 Department of Cell and Molecular Biology, Manipal School of Life Sciences, Manipal Academy of Higher Education, Manipal, Karnataka 576104 India

**Keywords:** Bulk multiomics, Epigenomics, Genomics, Granulosa cell, Metabolomics, Polyendocrine Metabolic Ovarian Syndrome, Proteomics, Single-cell multiomics, Theca cell, Transcriptomics

## Abstract

Polyendocrine metabolic ovarian syndrome (PMOS) is a heterogeneous disorder marked by a variety of clinical, hormonal, and metabolic disorders of an endocrine nature, affecting women of reproductive age and causing anovulatory infertility, metabolic dysfunction and hormonal disorders of a heterogeneous nature. Because of its complicated etiology and varied clinical presentation, PMOS’s underlying processes are still poorly understood despite its high incidence. There is a growing body of literature that attributes the multifactorial nature of PMOS to genetic predisposition, epigenetic modifications, environmental variables, and metabolic abnormalities, which result in dysregulation of multiple cellular pathways associated with ovarian function. Ovarian theca and granulosa cells are crucial to the pathophysiology of PMOS because alterations in their activity result in aberrant steroidogenesis, follicular arrest, reduced oocyte development, and disrupted endocrine signalling. Recent advances in high-throughput multiomics technology have greatly advanced our understanding of the molecular landscape of PMOS.

Recent advances in multiomics technologies, including genomics, epigenomics, transcriptomics, proteomics and metabolomics, have revolutionised our understanding of PMOS by allowing the integrated exploration of these molecular alterations. Genomic studies identified PMOS susceptibility loci, whereas epigenomic and transcriptomic studies revealed abnormal gene regulation and miRNA regulation. Proteomic and metabolomic profiling further elucidated functional disruption in protein networks and metabolic pathways, particularly in theca and granulosa cells.

Bulk multiomics approaches have substantially advanced our understanding of PMOS through the identification of genetic, epigenetic, transcriptomic, proteomic, and metabolic alterations associated with disease pathogenesis. Cellular function and heterogeneity are largely governed by complex gene regulatory networks (GRNs) and dynamic cell-to-cell communication mechanisms. Recent advances in single-cell multiomics approaches have enabled the identification of cell-specific molecular signatures and improved the understanding of the complex interactions between ovarian cell populations. Integration of bulk and single-cell multiomics datasets offers a more nuanced perspective on disease mechanisms. By integrating multiomics data, the present review aims to provide a more detailed understanding of the PMOS, thereby facilitating the identification of clinically significant biomarkers, the clarification of key regulatory pathways, and the identification of potential treatment targets. Collectively, these findings provide a framework for future translational research to improve the diagnosis and management of PMOS.

## Introduction

Polyendocrine metabolic ovarian syndrome (PMOS), previously known as polycystic ovary syndrome (PCOS) [1], is the most common endocrine and metabolic disorder in women of reproductive age. It is characterised by irregular menstruation, hyperandrogenism and polycystic ovarian morphology [2]. PMOS is not only a reproductive ailment, but also a syndrome with metabolic consequences that can negatively affect the health of women at multiple stages of reproductive and post-reproductive life [3]. Over time, expert consensus led to the development of the PMOS diagnostic criteria. It was formally recognised later after being first described by Stein and Leventhal, based on a triad of irregular menstruation, hirsutism, and enlarged ovaries with numerous follicles. Criteria for chronic anovulation and hyperandrogenism (clinical or biochemical) were established at a National Institutes of Health (NIH) conference in 1990. Later studies confirmed the use of ultrasound for ovarian evaluation. As a result, the Rotterdam criteria were developed, which included polycystic ovarian morphology and recognised PMOS as a spectrum condition in 2003. Any two of the three Rotterdam criteria, chronic anovulation, hyperandrogenism, or polycystic ovarian morphology, can now be included in the diagnostic criteria [4]. According to estimates, the prevalence of PMOS in women aged 17 to 45 varies from 8.2% to 22.5% in India and between 5.5% and 12.6% globally [5]. Geographical locations and ethnic groups have considerably different rates of PMOS incidence.

To improve clinical outcomes in the era of targeted therapeutics, drug discovery focuses on disease mechanisms and leverages extensive big data analysis. Owing to the large and diverse range of ‘omics’ data types, the field of biological research has entered a big data era. There is substantial interest in multiomics analysis, which involves the integration of data from several ‘omics’ levels. Investigating the relationships among the various ‘omics’ layers, such as genomics, transcriptomics, epigenomics, proteomics, and metabolomics, may yield important biological and therapeutic information. The ‘omics’ revolution allows for large-scale, economical biological quantification, starting with genomic sequencing and extending to next-generation sequencing (NGS) and mass spectrometry. Multiomics methods combine information from various methods, such as mass spectrometry (proteomics, metabolomics) and sequencing (genomics, transcriptomics, epigenomics), to provide a thorough picture of molecular activity [6]. The genotypic and phenotypic variability of bulk tissue can be determined using single-cell sequencing technology, which increases our understanding of the underlying mechanisms [7]. Although single-cell approaches provide unprecedented resolution of ovarian cell heterogeneity, their application in PMOS remains very limited and has yet to be explored.

Granulosa cells (GCs) and theca cells (TCs) are crucial for follicular formation, sex hormone production, and menstrual cycle regulation, and are essential for ovarian function. In folliculogenesis, GCs (mural GCs and cumulus cells) differentiate and produce estrogen from androgens from adjacent TCs. TCs, which are derived from the ovarian interstitial stroma, in turn respond to signals from growing follicles to take part in the generation of androgens. Coordinated actions of these cell types are critical for healthy reproductive physiology. Disruption of the complex processes in GCs and TCs may lead to infertility or serious endocrine disorders such as PMOS. Because of their intricate interactions, both cell types are crucial [8].

**Fig. 1:**
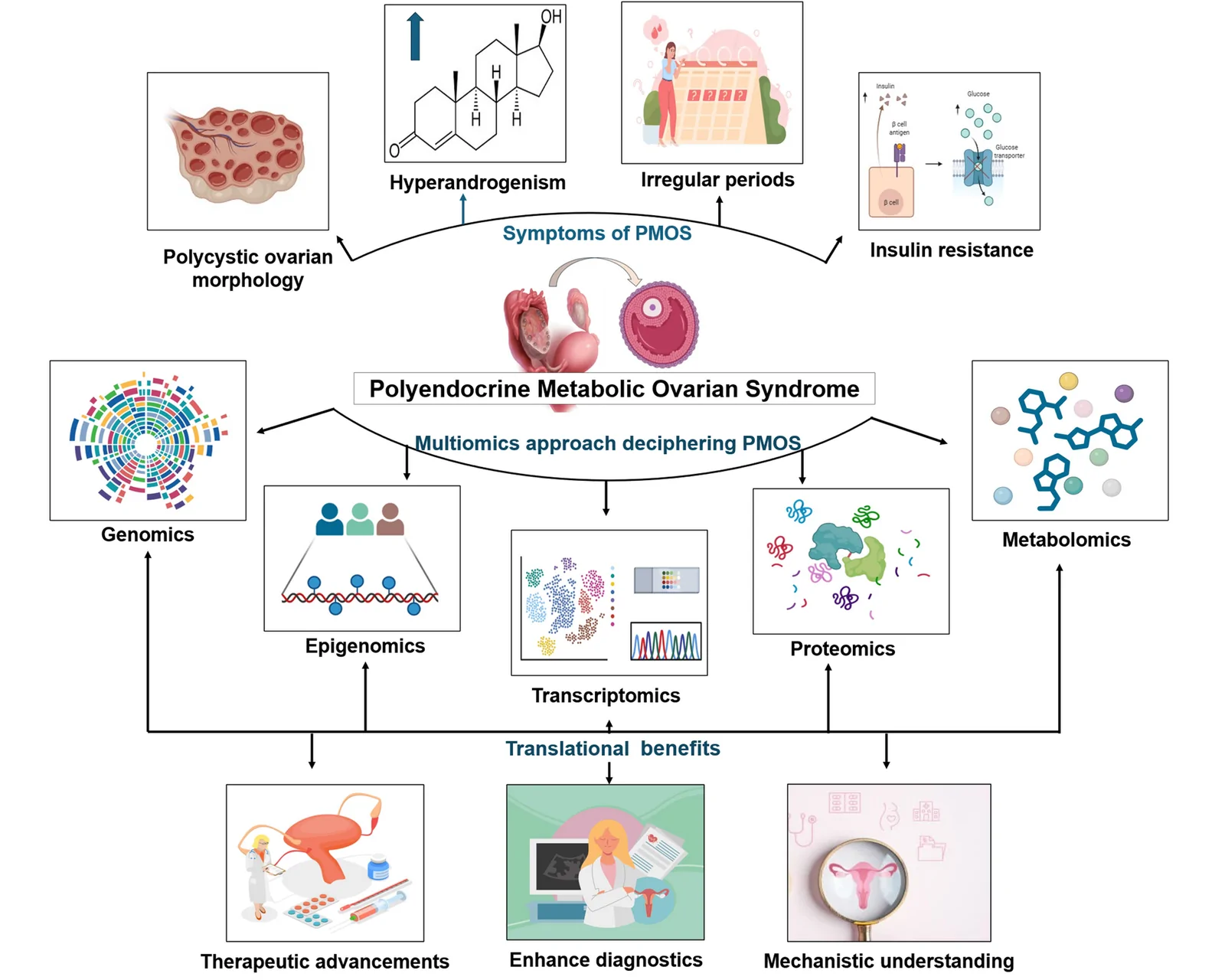
Overview of PMOS pathophysiology and the role of multiomics approaches in deciphering disease mechanisms. Schematic representation of the multi-omics strategy applied to elucidate the complex pathophysiology of PMOS. The clinical features of PMOS, including polycystic ovarian morphology, hyperandrogenism, menstrual irregularities, and insulin resistance, are linked to underlying molecular alterations. Integration of genomics, epigenomics, transcriptomics, proteomics, and metabolomics provides a comprehensive view of disease-associated genetic variation, regulatory mechanisms, altered molecular pathways, and metabolic changes. This integrated approach enhances understanding of PMOS biology and supports translational outcomes, including improved diagnostics, therapeutic development, and mechanistic insights into disease progression

Despite the considerable advances achieved through multiomics research, several challenges remain, including data heterogeneity, difficulties in integrating diverse omics layers, and limited coverage across biological systems. In addition, continued improvements in experimental and analytical methodologies will be essential for enhancing data quality, resolution, and interpretability. This review examines the possibilities provided by bulk single-cell analysis approaches in elucidating the underlying pathophysiology of PMOS from the perspective of multiomics fields, including genomics, epigenomics, transcriptomics, proteomics, and metabolomics and highlights the single-cell multiomics approaches to advance mechanistic understanding and translational research in PMOS, while also discussing the challenges associated with integrating multiomics data and the current limitations of these approaches. Finally, we identify key priorities and future directions to facilitate their effective application in research and clinical settings. (Refer to Fig. 1)

### Importance of bulk and single-cell multiomics approach in unravelling PMOS

Although advances in genotyping and data integration have led to the identification of many disease-associated genetic variants, much of the heritability remains unexplained. Both genetic and environmental variables influence common diseases, which frequently include regulatory changes rather than coding mutations. By connecting biomolecules with phenotypes and finding important pathways and biomarkers, multiomics integration aids in the discovery of causative mechanisms that go beyond simple correlations [9]. Additionally, it may help improve the understanding of molecular interactions, biological aging, and disease development and could support future applications in precision medicine and systems biology methodologies [10]. Broad variant discovery is made possible by whole-genome sequencing (WGS), while their role in diagnostics is facilitated by integrating data from various omics approaches, such as transcriptomics, proteomics, and metabolomics. Despite several limitations associated with experimental design, computational tools, data integration, and cost, multiomics improves the interpretation of variation pathogenicity, disease diagnosis and prognosis [11, 12]. To detect individual disease risk and monitor risks associated with it, Integrative Personal Omics Profiling (iPOP) combines information from the genome, transcriptome, proteome, and metabolome. According to the study performed by Chen et al., personalised omics profiling has been used successfully to detect healthy and diseased states, although it is still limited by its interpretability and high cost [13].

Bulk sequencing approaches enable molecular profiling at the cell-population level by capturing averaged signals derived from a large number of cells within a sample. In contrast, single-cell sequencing technologies allow the characterisation of thousands of individual cells simultaneously, providing high-resolution insights into cellular heterogeneity and cell-specific molecular states. Integrative analysis of multiomics datasets generated through both bulk and single-cell approaches offers a more comprehensive understanding of biological processes because it can capture many layers of molecular information within individual cells, including gene expression, chromatin accessibility, protein levels, and metabolites and can accelerate the translation of molecular discoveries into clinical applications [14]. Through the identification of disease-causing cell types and signalling pathways at the cellular level, this technique contributes immensely to the field of biomarker discovery and precision medicine for disorders such as PMOS [15, 16]. In essence, despite the present limitations, multiomics integration remains a cornerstone of systems and precision medicine with strong potential to improve diagnosis, prognosis, and personalised treatment [17]. This technique enables better biomarker identification and the quicker development of precision therapies because of the ability to preserve the cellular context and dynamics of interactions at the molecular level. Taking all the factors into account, multiomics is superior to traditional methods and represents an effective way to elucidate the complex biology of diseases with unprecedented accuracy.

### Role of theca cells and granulosa cells in PMOS pathogenesis

The ovary is an extremely well-organised microenvironment that combines different somatic cell types, such as granulosa, theca, and stromal cells, with germ cells, or oocytes. The intricacy of this structure allows important interactions that are required for folliculogenesis, which involves the formation of follicles, within which the oocytes are contained, along with their development and maturation, as well as the development of the oocyte and its surrounding somatic cells [18].

GCs interact with oocytes to create a favourable environment for folliculogenesis and oocyte maturation. These are crucial steroidogenic cells found in the ovarian follicle. In response to several stimuli, such as FSH, which stimulates aromatase to produce estrogen and, in concert with estrogen, activates the expression of the LH receptor, thereby promoting GC maturation, follicle growth, and fertility, these cells experience notable functional and molecular changes. When these cells malfunction, folliculogenesis and steroid hormone production are disrupted, which can lead to conditions such as PMOS [19].

Adjacent to the follicular basal lamina, TCs form the vascularised theca interna layer. They contain lipid vesicles that store cholesteryl esters, agranular endoplasmic reticulum, and numerous mitochondria, which are ultrastructural indicators of steroidogenesis. These cells specialise in the production of steroid hormones, mainly androgens, which are made possible by endoplasmic reticulum and mitochondrial enzymes. The two-cell, two-gonadotropin model is essential for ovarian estrogen biosynthesis. GCs (FSH-stimulated) have an abundance of aromatase to convert the androgens derived from TC into estrogen, while TCs (via CYP17, LH-stimulated) produce androgens but lack aromatase (*CYP19*). The degree of aromatase expression in TCs varies greatly between species [20]. Ovarian estrogen biosynthesis is explained by the two-cell, two-gonadotropin theory of Armstrong et al., which uses cellular compartmentalisation. The first functional compartment is theca cells, which produce androgens when stimulated by LH and have LH receptors. *CYP19* catalyses the conversion of theca-derived androgens into estrogen by stimulating granulosa cells, which are found in the second compartment. These cells have FSH receptors. This model is supported by the fact that FSH receptors are specifically localised to granulosa cells and LH receptors to theca cells. FSH and LH work in concert, but it is challenging to conclusively link each hormone to in vivo steroidogenesis because there are few clinical conditions associated with total gonadotropin deficiency, and because gonadotropins were previously difficult to separate before recombinant technology, which produced preparations that contained both hormones [21].

## Dysregulations in theca cells and granulosa cells revealed by bulk multiomics approaches in PMOS

### Functional genomics and epigenomics insights into theca cell and granulosa cell dysfunction

Our understanding of the root causes of PMOS has significantly improved as a result of advances in genomics research. These genomic discoveries are essential for revealing the complex nature of PMOS and suggesting novel lines of inquiry. Acquiring cell-specific gene expression and functional genomics data from TCs and GCs is still challenging, although the genome is mostly conserved across cell types. Studies specifically investigating genomics in ovarian TCs and GCs remain limited.

Selective deletion of *PTEN* in theca interstitial (TI) cells leads to infertility, irregular estrous cycles, increased androgen production, and upregulation of *LHCGR* and steroidogenic genes. Overactivation of the PI3K-AKT1 signalling pathway causes this phenotype by phosphorylating and inactivating *FOXO1*, which releases its inhibition of *LHCGR* transcription. The resulting increase in *LHCGR* strengthens LH-mediated signalling, creating a positive feedback loop that increases androgen production even more. As a result, persistent PI3K-AKT-FOXO1 axis activation in PTEN-deficient TCs recapitulates important aspects of hyperandrogenism and ovarian dysfunction linked to PMOS [22].

Although genetic changes provide important information about the molecular basis of PMOS, genetic susceptibility alone is unlikely to fully explain the heterogeneity and complex clinical manifestations of the syndrome. There is increasing evidence that the environment and hormones interact with genetic susceptibility to influence ovarian cell function and disease progression through epigenetic mechanisms. Therefore, epigenomic studies have become an important approach for understanding the effects of external stimuli on the patterns of gene expression in TC and GC without alteration to the DNA sequence.

Our understanding of PMOS is significantly improved by an epigenomic approach, which examines how gene expression is regulated without altering the DNA sequence itself. Examples of this include DNA methylation, which may either activate or inactivate genes, and non-coding RNA, such as miRNA regulation, which controls protein levels following transcription. Developmental epigenetic programming, in which exposure to unfavourable hormonal conditions during crucial developmental windows results in long-lasting changes in gene regulation, is one of the earliest pathways linked to PMOS pathogenesis.

Epigenetic modifications, particularly DNA methylation, are associated with the pathophysiology of PMOS and may stem from prenatal effects. According to growing research, exposure to androgens during pregnancy may cause PMOS through long-lasting epigenetic changes that affect gene expression. Studies in prenatally androgenised rat models have demonstrated altered DNA methylation patterns in ovarian TCs, particularly in the expression of steroidogenic genes such as *CYP17*, *GATA6*, and *StAR*. Increased gene expression has been linked to hypomethylation at particular CpG sites in the promoters of *GATA6* and *StAR*, indicating that androgen exposure during crucial developmental windows may increase steroidogenic activity. As *GATA6* controls downstream targets, such as *CYP17*, its altered methylation offers a molecular explanation for persistent androgen excess in PMOS [23]. These results imply that persistent steroidogenic dysregulation may have a biological basis established by androgen-mediated epigenetic programming, predisposing ovarian cells to the hyperandrogenic phenotype typical of PMOS.

In addition to developmental programming, aberrant DNA methylation patterns in GCs are also indicative of epigenetic changes that directly affect follicular development and ovarian function. The GCs that are essential in folliculogenesis and oocyte development are affected not only by developmental programming but also by epigenetic dysregulation. Compared with controls, PMOS patients have different GC methylation profiles, including global hypermethylation. Disruptions in gene expression and in follicular growth occur because of increased methylation of *RBP4* and decreased methylation of the *SPP1*,* IL12B*, and *F2R* genes [24]. This finding is corroborated by the discovery of 106 CpG positions whose methylation patterns differed between PMOS patients and controls by epigenome-wide association studies (EWAS) conducted using ovarian granulosa-lutein cells. Numerous of these locations are connected to genes that regulate metabolism and ovarian function, including loci linked to PMOS susceptibility, such as *BMPR1A*,* FERMT2*, and *HMGA2*. These results provide a consistent epigenetic signature across PMOS subgroups and are corroborated by pyrosequencing [25]. Taken together, these methylation anomalies suggest that epigenetic dysregulation in GC extends beyond specific genes and could be a more comprehensive cause of PMOS follicular dysfunction.

Crucially, epigenetic changes are not just static developmental events but may be sustained by the hyperandrogenic environment of the PMOS. Hyperandrogenism, which leads to epigenetic alterations in ovarian cells, is among the most important features of PMOS. Elevated androgen levels promote GC dysfunction and apoptosis in a dose-dependent manner, leading to impaired follicular development and follicular arrest in PMOS patients. Target gene promoters become hypomethylated as a result of androgen-mediated downregulation of the DNA methyltransferases *DNMT1*,* DNMT3A*, and *DNMT3B*. Under these circumstances, GC apoptosis is promoted by the upregulation of one such gene, *PDCD4*. Hormonal imbalance and cellular dysfunction in PMOS are directly linked, as demonstrated by the androgen-mediated epigenetic control of *PDCD4* [26]. In a similar vein, *YAP1* is epigenetically dysregulated in PMOS and has been identified as a susceptibility gene in genome-wide association studies. Increased mRNA and protein expression in ovarian GCs is correlated with decreased *YAP1* promoter methylation. Interestingly, testosterone reduces *YAP1* methylation in a dose-dependent way, providing more evidence that androgen-mediated epigenetic regulation might be involved in the development of PMOS [27]. These findings support a self-perpetuating cycle in which hyperandrogenism promotes epigenetic dysregulation, consequently worsening ovarian dysfunction and androgen excess in PMOS.

In addition to the role of DNA methylation in the pathogenesis of PMOS, non-coding RNAs such as microRNAs (miRNAs) have been implicated in controlling ovarian function through the regulation of steroidogenesis, GC proliferation and apoptosis. In PMOS, several miRNAs are dysregulated, which contribute to the disease progression via different but related mechanisms. For example, miR-486-5p, which typically targets *MST4* kinase to prevent GC growth, is markedly downregulated in PMOS tissues [28]. Similar to this, miR-16 plays a direct role in regulating PDCD4 for controlling GCs’ survival. In hyperandrogenic circumstances, its downregulation results in increased apoptosis and decreased cell cycle progression [29]. On the other hand, miR-320a, which targets *PCGF1* and is increased in an insulin-dependent way, increases GC proliferation while preventing apoptosis, connecting ovarian disorders to metabolic failure [30]. These miRNAs are involved in the loss of GC homeostasis in PMOS through the regulation of proliferation, apoptosis and insulin-responsive pathways.

In addition to apoptosis induction, cell death pathways play another major role in the effects of miRNAs. The miR-200 family member miR-141-3p, which typically inhibits apoptosis by targeting *DAPK1*, is downregulated in PMOS. Its decreased expression causes increased GC apoptosis [31]. Conversely, miR-93-5p is upregulated and promotes both apoptosis and ferroptosis by suppressing *GPX4*, *SLC7A11*, and *Nrf2*, and inhibiting the NF-κB signalling pathway. These effects collectively contribute to GC loss and ovarian dysfunction, further emphasising the role of miRNA-mediated epigenetic dysregulation in PMOS [32]. Therefore, miRNA-mediated modulation of apoptosis and ferroptosis may represent an important mechanism contributing to follicular arrest and progressive ovarian dysfunction in PMOS.

Epigenetic dysregulation in the PMOS is not restricted to the GC but also affects the TC, where it directly influences androgen biosynthesis. It has been demonstrated that the shortened version of the PMOS-associated gene *DENND1A.V2* is regulated by decreased expression of miR-130b-3p. This change increases androgen production in PMOS by activating downstream signalling pathways such as MAPK1, PI3K, and cAMP/PKA. This results in the overexpression of important steroidogenic enzymes such as *CYP17A1*,* CYP11A1*, and *HSD3B2* [33]. These findings provide mechanistic evidence that altered miRNA expression contributes directly to the excessive androgen production that defines the PMOS phenotype. (Refer to Table 1.)

**Table 1:** Summary of miRNA epigenomics findings in PMOS

Potential functional impact in PMOS	Key miRNAs involved	Major gene targets	Reference
Increased granulosa cell proliferation	miR-320a	*PCGF1*	[30]
Decreased granulosa cell proliferation	miR-486-5p	*MST4*	[28]
Granulosa cell apoptosis	miR-16, miR-141-3p, miR-93-5p	*PDCD4*,* DAPK1*,* GPX4*,* SLC7A11*,* Nrf2*	[29, 31, 32]
Granulosa cell ferroptosis	miR-93-5p	*GPX4*,* SLC7A11*,* Nrf2*	[32]
Increased androgen biosynthesis in theca cells	miR-130b-3p	*DENND1A.V2*	[33]

Long non-coding RNAs (lncRNAs) further add to the complexity of epigenetic regulation in the PMOS by acting as competitive endogenous RNAs (ceRNAs) that modulate miRNA activity. High-throughput sequencing studies have revealed several lncRNAs that are significantly dysregulated in PMOS and are associated with pathways related to immune response, lipid metabolism, and steroid hormone biosynthesis. These genes include genes involved in GC proliferation and metabolic regulation, such as *EREG*, *YAP1*, and *ENTPD6* [34]. One well-characterised regulatory axis involves lncRNA ZFAS1, which is upregulated in PMOS and functions by sponging miR-129, thereby increasing HMGB1 expression. Functional studies have demonstrated that silencing ZFAS1 or restoring miR-129 enhances GC proliferation, reduces apoptosis, and improves hormone secretion, underscoring the importance of lncRNA–miRNA interactions in PMOS pathophysiology [35]. Together, these findings highlight the role of lncRNA-mediated regulatory circuits in the integration of proliferative, metabolic, inflammatory, and steroidogenic signals within the PMOS ovary.

Together, these results suggest that epigenetic dysregulation is a key mediator connecting genetic susceptibility, hormonal and environmental factors, and ovarian dysfunction in patients with PMOS. Disruption of steroidogenesis, folliculogenesis, insulin signalling, apoptosis and inflammatory pathways through coordinated changes in DNA methylation, miRNA expression and lncRNA-mediated regulatory networks. These molecular changes contribute significantly to hyperandrogenism, follicular arrest, ovulatory dysfunction and other characteristic manifestations of PMOS and underscore the central role of epigenetic mechanisms in disease pathogenesis.

### Transcriptomics and functional transcriptomics insights into theca cell and granulosa cell dysfunction

Transcriptomics is a powerful tool for understanding the intricacies of the PMOS. By profiling the entire collection of RNA transcripts present in cells or tissues, this technique offers a comprehensive understanding of gene expression patterns. The identification of genes that are differentially expressed in key tissues such as the ovary, particularly in the granulosa and theca cells, which support the syndrome characteristics, has made transcriptomic studies essential to PMOS research. Collectively, the results of the transcriptomic analyses provide a functional bridge between upstream genetic and epigenetic alterations and the downstream cellular abnormalities observed in PMOS.

Among the transcriptomic alterations linked to genetic susceptibility, DENND1A has emerged as one of the most extensively studied PMOS-associated genes. Exonization of intronic regions within intron 20 produces a distinct exon (20 A) of *DENND1A.V2* in PMOS TCs. Both the V1 isoform and V2 isoform are expressed by human adrenal NCI-H295A cells, and overexpression of V2 increases the expression of steroidogenic genes, such as *CYP17A1* and *CYP11A1*, as observed in TCs. Several GWAS-identified SNPs inside DENND1A are linked to PMOS risk, even though sequence variants close to exon 20 A do not always increase V2 splicing. Significantly, *DENND1A.V2* is overexpressed in PMOS TCs, where it causes a steroidogenic phenotype. These effects are reversed when *DENND1A.V2* is knocked down, indicating that dysregulated alternative splicing plays a role in enhanced androgen production in PMOS [36]. These observations provide direct evidence that altered transcript processing can translate genetic susceptibility into a steroidogenic phenotype.

Further mechanistic studies have clarified how DENND1A.V2 contributes to androgen excess. *DENND1A.V2* mainly increases the expression of *CYP17A1* to boost androgen production. Reduced levels of the transcriptional corepressor ZNF217 may promote this effect by increasing the expression of *DENND1A.V2*. Additionally, *DENND1A.V2* seems to operate within a relatively large regulatory network that includes additional PMOS-associated genes, including *LHCGR* and *RAB5B*, all of which contribute to hyperandrogenism [37]. In support of this, DENND1A.V2 colocalises with LHCGR at the plasma membrane and with RAB5B in endocytic vesicles and the perinuclear region, indicating a role in receptor trafficking and signalling. Upon stimulation with human chorionic gonadotropin (hCG) or forskolin, *DENND1A.V2* is translocated to the nucleus, suggesting that it plays an additional role in the transcriptional regulation of steroidogenic genes. Together, these findings highlight a coordinated functional network involving *DENND1A.V2*,* LHCGR*, and *RAB5B* drive aberrant androgen biosynthesis in PMOS [38]. Therefore, it seems that DENND1A.V2 is part of a coordinated regulatory network that increases the production of androgens in the ovaries.

In addition to the alternatively spliced V2 isoform, the full-length DENND1A transcript contributes to PMOS pathogenesis. Women with PMOS have overexpressed full-length *DENND1A (V1)* in their ovarian GCs. *DENND1A* overexpression decreases ovarian sensitivity to gonadotropins, hinders follicular growth, and increases testosterone synthesis, according to functional investigations conducted on transgenic mouse models. Mechanistically, *DENND1A* reduces receptor availability at the cell surface and decreases FSH responsiveness by promoting the internalisation and retention of the FSH receptor *(FSHR)* within endosomal and lysosomal compartments. Interestingly, FSH sensitivity in GCs is restored by *DENND1A* knockdown. *DENND1A.V1* is a major susceptibility gene that contributes to both reproductive and metabolic dysfunction in PMOS, as indicated by its positive correlation with blood testosterone levels and impaired ovarian function [39]. These findings indicate that DENND1A contributes not only to hyperandrogenism but also to impaired follicular responsiveness and ovarian dysfunction.

While DENND1A exemplifies how genetic susceptibility can influence ovarian function through altered transcript processing, transcriptomic studies have also revealed broader dysregulation of signalling networks that govern cellular proliferation, metabolism, and follicular development. A total of 1,114 differentially expressed genes linked to follicular development have been found in transcriptomic analysis of TCs in the PMOS rat model, with enrichment in important signalling pathways such as MAPK and PI3K–Akt. The upregulation of hub genes, including *mTOR*,* Akt*, and *CCND1*, indicates that overactivation of these pathways may drive abnormal TC proliferation and follicular dysfunction [40]. In support of this, altered PI3K–Akt signalling is also observed in GCs, where increased expression of miR-133a-3p inhibits this pathway, contributing to local ovarian insulin resistance and dysregulation of downstream targets such as *GLUT4*, *GSK-3β*, and *FOXO1* [41]. Furthermore, metabolic reprogramming has been demonstrated in PMOS models, in which NMN supplementation improves glycolysis by upregulating important enzymes (HK2, PKM2, LDHA) and modifying the PI3K–Akt, PPAR signalling, and steroidogenesis pathways to restore ovarian function [42]. These results imply that both the increased proliferation of ovarian cells and the metabolic abnormalities typical of PMOS are caused by inappropriate activation of signalling networks.

Transcriptomic investigations also revealed changes in intercellular communication systems, which are critical for optimal ovarian function, in addition to traditional growth-factor routes. Purinergic pathways further demonstrate changes in cell signalling. Functional P2Y receptors, especially *P2Y2R*, which mediates ATP/UTP-induced proliferation via ERK signalling, are expressed by ovarian TCs. TCs’ proliferation is disturbed in PMOS models due to the downregulation of *P2Y2R*, which is partially caused by higher androgen levels. These findings suggest that altered purinergic signalling plays a role in ovarian dysfunction [43]. Therefore, another way in which androgen excess disturbs ovarian homeostasis in PMOS could be through compromised purinergic signalling.

The consequences of altered signalling are further reflected in widespread transcriptional changes affecting key functional pathways within GC. Transcriptomic profiling of GCs in PMOS showed dysregulation of genes related to lipid metabolism, steroidogenesis, and inflammation. Genes including *PNPLA3*,* MVD*,* PCSK9*,* MMP9*,* OSM*,* LCK*,* TREM1*, and *C3* were mainly upregulated, leading to impaired oocyte development capacity, oocyte maturation, and ovarian dysfunction. While *FADS2* was downregulated, indicating altered cholesterol metabolism and ovarian dysfunction [44]. Collectively, these transcriptional changes suggest that poor follicular growth and ovarian dysfunction are directly caused by disturbed lipid metabolism, steroidogenesis, and inflammatory reactions.

Transcriptomic investigations have demonstrated the significance of regulatory RNAs in influencing GC behavior in addition to protein-coding genes. Targeting *ESR2*, miR-186 and miR-135a are elevated in PMOS GCs. This activates the ESR1–p53 pathway and increases *CDKN1A* expression, which promotes proliferation and inhibits apoptosis [45]. Other miRNAs, such as miR-6881-3p, impair GC proliferation and steroidogenesis by targeting *SMAD4*, affecting TGF-β signalling and gonadotropin responsiveness [46]. On the other hand, miR-424 controls GC proliferation by modifying Apelin/APJ signalling, whereas decreased expression of miR-324-3p increases GC proliferation and death via the WNT2B axis [47, 48]. These miRNAs significantly contribute to GC dysfunction in the PMOS by regulating cell-cycle progression, apoptosis, steroidogenesis, and gonadotropin responsiveness.

According to new transcriptome data, PMOS disease may also be influenced by disruptions in cellular stress-response pathways. Emerging evidence from a study revealed that *NQO1*,* ATF3*,* LPIN1*,* BNIP3*,* DDIT4*,* NOS2*,* SLC2A1*, and *SLC2A6* were significantly downregulated in PMOS GCs, indicating altered ferroptosis regulation. A number of these genes are associated with parameters that affect the quality of the egg, indicating their involvement in cases of PMOS-associated infertility [49]. These findings link oxidative stress and ferroptosis dysregulation to reduced oocyte quality and infertility associated with PMOS. Thus, transcriptomic alterations extend beyond proliferation and steroidogenesis to encompass cellular stress-response pathways that may directly affect reproductive outcomes.

Interactions between metabolic, epigenetic, and hormonal regulatory pathways also impact transcriptomic dysregulation in PMOS. Overexpression of *KAT2B* reduces aromatase (*CYP19A1*) expression via histone acetylation and alters pyruvate metabolism, impairing follicular development [50]. In addition, *CYP19A1* expression and estrogen production are influenced by androgen receptor (AR) interactions with *FOXL2*, which connects hormonal imbalance to transcriptional dysregulation in PMOS [51]. GC dysfunction and follicular arrest are thus worsened by changes in chromatin regulation and androgen-responsive transcriptional networks. These investigations have elucidated the dysregulated pathways that contribute to the pathophysiology of PMOS, including steroidogenesis, folliculogenesis, inflammation, and insulin signalling.

Collectively, transcriptomic studies demonstrate how genetic susceptibility and epigenetic influences are translated into functional molecular alterations within TC and GC. Abnormal cell proliferation, poor folliculogenesis, hyperandrogenism, and metabolic dysfunction are caused by the dysregulation of signalling pathways, regulatory RNAs, metabolic networks, steroidogenic genes, and stress-response mechanisms. These results identify potential targets for future therapeutic intervention and establish transcriptomics as a crucial mechanistic link between upstream genetic changes and the clinical symptoms of PMOS. (Refer to Table 2.)

**Table 2:** Summary of transcriptomics findings in PMOS

Functional pathway/process	Key genes/miRNAs	Biological consequence in PMOS	Reference
MAPK and PI3K–Akt signalling	*mTOR*,* AKT*,* CCND1*,* GLUT4*,* GSK-3β*,* FOXO1*	Abnormal theca cell proliferation, follicular dysfunction, ovarian insulin resistance	[40, 41]
Purinergic signalling	*P2Y2R*	Reduced theca cell proliferation and ovarian dysfunction	[43]
Lipid metabolism, steroidogenesis, and inflammation	*PNPLA3*,* MVD*,* PCSK9*,* MMP9*,* OSM*,* LCK*,* TREM1*,* C3*,* FADS2*	Impaired oocyte maturation, altered cholesterol metabolism, ovarian dysfunction	[44]
Increased ESR1–p53 pathway, TGF-β signalling, Apelin/APJ signalling	miR-186, miR-135a, miR-6881-3p, miR-424, miR-324-3p	Altered granulosa cells proliferation, apoptosis, steroidogenesis, and gonadotropin responsiveness	[45–47]
Ferroptosis and oxidative stress	*NQO1*,* ATF3*,* LPIN1*,* BNIP3*,* DDIT4*,* NOS2*,* SLC2A1*,* SLC2A6*	Reduced oocyte quality and PMOS-associated infertility	[49]

### Proteomics and metabolomics insights into theca and granulosa cell dysfunction

Proteomics and metabolomics provide functional insight into PMOS by revealing how upstream genetic, epigenetic, and transcriptomic alterations are ultimately translated into changes in protein expression, signalling pathways, and cellular metabolism. These methods help explain how changes in TC and GC activity lead to the PMOS phenotype and capture the downstream molecular effects of ovarian dysfunction.

A central functional consequence of ovarian dysfunction in PMOS is the establishment of a hyperandrogenic follicular microenvironment. Recent research has shown for the first time that intrafollicular androgen levels produced by TCs are selectively increased in PMOS, supporting the concept that ovarian hyperandrogenism plays a key role in the disorder’s pathophysiology. Moreover, significant correlation was found between follicular androgen levels and several adipokines, including apelin, chemerin, resistin and omentin. These results suggest a significant interaction between androgen signalling and adipokine regulation in the ovarian microenvironment. These interactions may be important in ovarian function and the development of PMOS [52]. These results imply that androgen excess is intimately linked to endocrine and metabolic signalling in the ovarian follicle, which advances the disease.

The impact of this altered ovarian environment is further reflected in profound metabolic reprogramming within GCs. A metabolomic study revealed that TP53-induced glycolysis and apoptosis regulator (TIGAR) overexpression affects several metabolic pathways in PMOS GCs, including those associated with energy metabolism, such as the TCA cycle, oxidative phosphorylation, mTOR signalling, and glycolysis. The core metabolites of the metabolic network, such as niacinamide, L-tyrosine, and L-tryptophan, were markedly elevated. These metabolic disturbances contribute to impaired GC function and PMOS progression by disrupting cellular energy homeostasis and ovarian physiology [53]. Similarly, in another study, reduced pyruvate levels were among the metabolomic changes in the PMOS follicular microenvironment, which are indicative of compromised glycolytic activity and disturbed oocyte GC metabolic coupling [54]. Thus, disruption of cellular energy metabolism appears to be a key mechanism underlying GC dysfunction in PMOS.

Proteomic research has shown extensive changes in signalling and stress-response pathways that are consistent with these metabolic disruptions. DHEAS exposure changed the expression of several proteins in PMOS patients’ GCs, according to proteomic research. The study found that cumulus GCs had reduced expression of a number of proteins, most likely as a result of the WNT signalling pathway being downregulated. On the other hand, it was discovered that DHEAS-treated PMOS cells had increased or overexpressed levels of proteins such as DENND1A, THADA, NAMPT, GSTs, PKD1, HIF-1, and immune-related proteins. Important signalling pathways were also triggered, including VEGF, TGF-β, PI3K, MAPK, and Toll receptor signalling. The pathophysiology of PMOS may be influenced by increased androgen production, insulin resistance, inflammation, oxidative stress, and ovarian dysfunction, according to these proteome changes [55]. Collectively, these proteomic changes indicate that excess androgen drives broad molecular alterations affecting steroidogenesis, insulin sensitivity, inflammation, and ovarian function.

Several proteomic investigations have further identified specific molecular regulators that may directly influence ovarian homeostasis. According to proteomic studies, FDPS expression was markedly downregulated in the GCs and ovarian tissues of PMOS humans and DHEA-induced PMOS mice. In the context of PMOS, decreased granulosa cell proliferation, follicular dysplasia, and ovarian dysfunction were linked to lower FDPS levels. Additionally, the study found that PMOS ovaries had lower RAC1 expression and activity, which suggests that the RAC1/MAPK/ERK signalling pathway is suppressed. In PMOS mice, overexpression of FDPS alleviated metabolic and ovarian problems, enhanced granulosa cell proliferation, and triggered RAC1/MAPK/ERK signalling. These results imply that whereas FDPS restoration protects ovarian function, downregulation of FDPS and RAC1 contributes to the development of PMOS [56]. These findings suggest that disruption of FDPS-mediated signalling contributes to ovarian dysfunction, whereas restoration of this pathway may exert protective effects.

Beyond individual signalling molecules, proteomic profiling has uncovered extensive alterations in pathways governing cellular growth and metabolism. The proteomic analysis of GCs from PMOS patients identified 174 differentially expressed proteins, including 7 upregulated and 167 downregulated proteins. TOP2A and SPHKAP were discovered to be highly elevated in PMOS, indicating altered sphingolipid signalling and increased GC proliferation. On the other hand, PMOS GCs showed marked downregulation of FGF2, APOA2, GM2A, and MRPS16. These changed proteins were primarily enriched in ribosomal pathways, oxidative phosphorylation, cholesterol metabolism, and mitochondrial activity, suggesting disruptions in granulosa cell survival, follicular growth, energy metabolism, and lipid metabolism in PMOS [57]. Together, these alterations point to widespread disturbances in mitochondrial function, lipid metabolism, and follicular development in PMOS.

In addition to intracellular signalling abnormalities, proteomic studies highlight an important role for inflammatory and metabolic regulators. The differentially expressed proteins in GCs were mostly linked to lipid metabolism and inflammation, indicating that they might be involved in oocyte development during assisted reproductive technologies (ART). According to a study, insulin resistance, inflammation, and aberrant lipid metabolism were linked to AZGP1 (ZAG), which was markedly downregulated in PMOS. On the other hand, obesity, insulin resistance, inflammation, and dysregulated adipocyte differentiation were associated with CCN4 (WISP1), which was increased in PMOS, especially in obese PMOS patients. A1BG and APOC1, two other inflammatory proteins, were also altered in PMOS, suggesting problems with the metabolism of fats and carbohydrates [58]. These observations reinforce the close relationship between inflammation, insulin resistance, metabolic dysfunction, and reproductive abnormalities in PMOS.

Post-translational protein modifications provide an additional layer of functional regulation in PMOS. A quantitative acetyl-proteomic study revealed significant alterations in protein lysine acetylation in ovarian GCs, particularly in enzymes involved in critical metabolic pathways, such as glycolysis, fatty acid oxidation, BCAA catabolism, and the TCA cycle. It has been demonstrated that the mitochondrial enzyme ACAT1 is more acetylated, which may reduce its activity and impede ovarian follicles’ capacity to sustain metabolic homeostasis. Such metabolic alterations may be the cause of reduced egg quality and poor embryo development, which are commonly observed in PMOS [59]. Therefore, altered protein acetylation may represent a mechanistic link between metabolic dysfunction and impaired oocyte competence in PMOS.

The proteomic and metabolomic studies have shown how upstream molecular anomalies lead to functional changes in ovarian cells. Impaired GC and TC function, follicular arrest, and hyperandrogenism are caused by dysregulation of steroidogenesis, energy metabolism, inflammatory signalling, oxidative stress responses, mitochondrial activity, and post-translational protein changes. These discoveries establish proteomics and metabolomics as the downstream functional layer of PMOS pathogenesis, offering vital information about how molecular changes eventually show up as clinical illness and ovarian dysfunction. Understanding these molecular alterations provides deeper insight into ovarian dysfunction in PMOS. (Refer to Table 3.)

**Table 3:** Summary of proteomics and metabolomics findings in PMOS

Functional pathway/process	Key proteins/metabolites	Major alteration in PMOS	Reference
Increased androgen signalling and adipokine regulation	Intrafollicular androgens, apelin, chemerin, resistin, omentin	Enhanced androgen signalling and ovarian endocrine dysfunction	[52]
TCA cycle, oxidative phosphorylation, mTOR signalling, and glycolysis	TIGAR, niacinamide, L-tyrosine, L-tryptophan	Impaired granulosa cell energy homeostasis and ovarian physiology	[53]
Induced VEGF, TGF-β, PI3K, MAPK, and Toll receptor signalling	DENND1A, THADA, NAMPT, GSTs, PKD1, HIF-1	Hyperandrogenism, insulin resistance, inflammation, and ovarian dysfunction	[55]
Suppressed RAC1/MAPK/ERK signalling	FDPS, RAC1	Decreased granulosa cell proliferation, follicular dysplasia, and ovarian dysfunction	[56]
Enriched ribosomal pathways, oxidative phosphorylation, cholesterol metabolism, and mitochondrial activity	TOP2A, SPHKAP, FGF2, APOA2, GM2A, MRPS16	Impaired follicular growth, granulosa-cell survival, and lipid metabolism	[57]
Inflammation and metabolic dysfunction	AZGP1 (ZAG), CCN4 (WISP1), A1BG, APOC1	Insulin resistance and obesity-associated inflammation	[58]

**Fig. 2:**
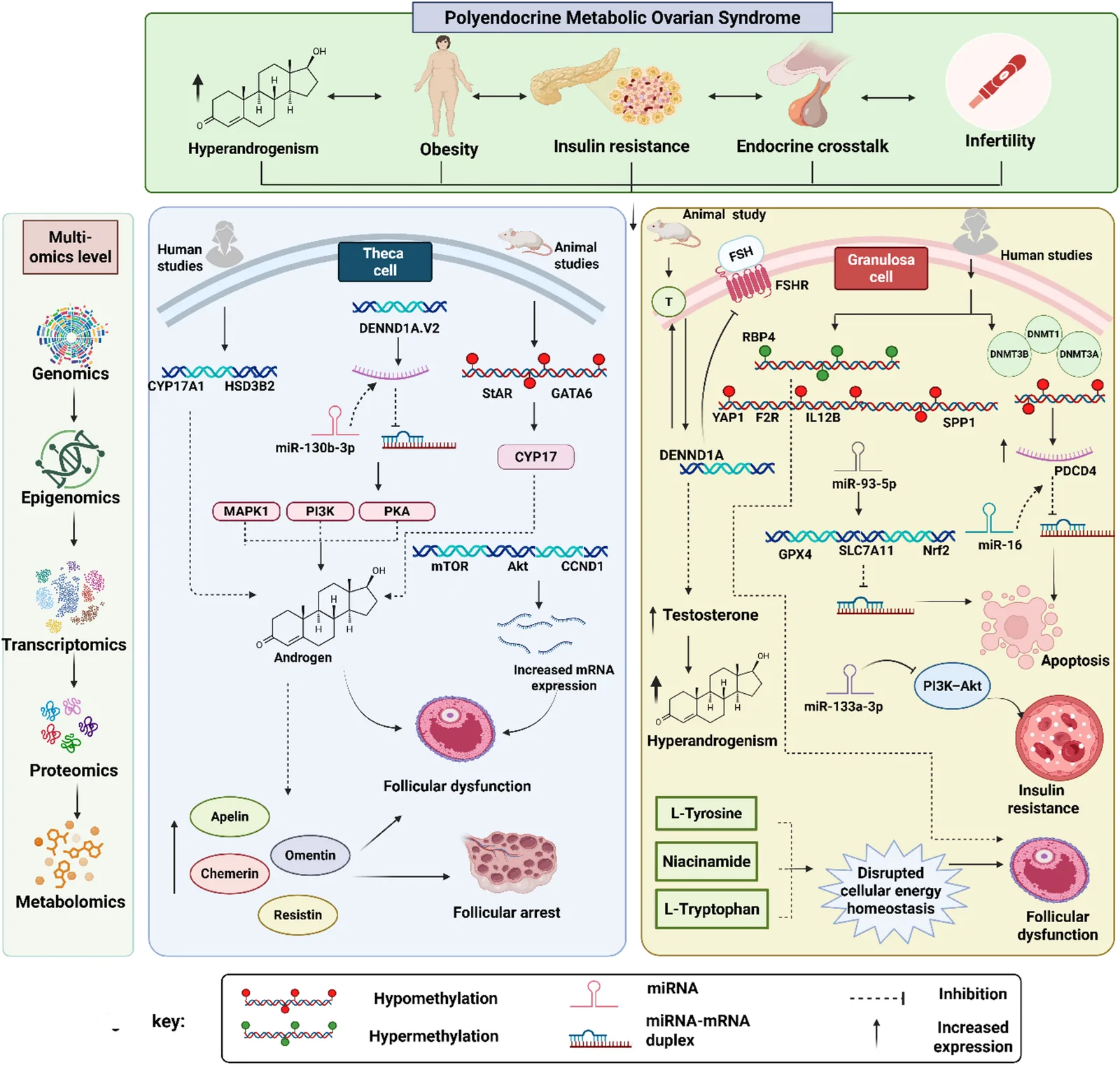
Bulk multiomics regulation of theca and granulosa cell dysfunction in PMOS. Schematic representation of the molecular mechanisms underlying PMOS through integration of multi-omics datasets across ovarian theca and granulosa cells. PMOS-associated systemic abnormalities, including hyperandrogenism, obesity, insulin resistance, endocrine imbalance, and infertility, influence ovarian cellular function. Genomic, epigenomic, transcriptomic, proteomic, and metabolomic layers contribute to the regulation of key molecular pathways involved in ovarian dysfunction. In theca cells, genetic and epigenetic alterations regulate steroidogenic pathways involving genes such as CYP17A1, HSD3B2, STAR, and GATA6, leading to increased androgen biosynthesis and follicular dysfunction. Epigenetic modifications, such as DNA methylation changes, influence steroidogenic gene activity, while miRNA-mediated regulation (e.g., miR-130b-3p) modulates DENND1A.V2. This change increases androgen production in PMOS by activating downstream signalling pathways like MAPK1, PI3K, and cAMP/PKA, thereby increasing testosterone production. Adipokines (apelin, chemerin, omentin, and resistin) are positively associated with androgen excess, promote follicular dysfunction and follicular arrest, contributing to the PMOS phenotype. In granulosa cells, altered DNA methylation, miRNA-mediated regulation, and transcriptional changes involving genes such as FSHR, DENND1A, YAP1, F2R, IL12B, and SPP1 contribute to impaired follicular maturation and follicular dysfunction. DENND1A overexpression increases testosterone synthesis. Upregulated miR-93-5p promotes apoptosis of granulosa cells by suppressing GPX4, SLC7A11, and Nrf2. miR-133a-3p alters PI3K–Akt signalling, contributing to insulin resistance. Niacinamide, L-tyrosine, and L-tryptophan are markedly elevated and contribute to impaired GC function and PMOS progression by disrupting cellular energy homeostasis and ovarian physiology

Overall, bulk multiomics approaches have provided a comprehensive view of the molecular landscape underlying PMOS by integrating genetic, epigenetic, transcriptomic, proteomic, and metabolomic alterations. These approaches have identified key dysregulated pathways involved in steroidogenesis, insulin signalling, inflammation, follicular development, and metabolic dysfunction. (Refer to Fig. 2.)

## Single-cell transcriptomics in theca cells and granulosa cells in the PMOS

Single-cell omics approaches have revolutionised the understanding of cellular heterogeneity by enabling molecular profiling at the individual cell level. Unlike bulk multiomics, which provides averaged molecular signatures from a population of cells, single-cell technologies allow identification of distinct cell populations, cell-specific regulatory programs, and disease-associated cellular states. In PMOS, single-cell studies have focused on transcriptomic profiling, particularly single-cell RNA sequencing (scRNA-seq), to characterise alterations in ovarian cell populations such as GC and TC.

GC analysis revealed an increased population of KRT8^high^ mural GCs with elevated expression of KRT8/KRT18, genes associated with granulosa cell differentiation, survival, and follicular development, potentially contributing to the increased follicle number observed in PMOS. Theca cell populations, including theca interna and theca externa cells, showed increased abundance with enhanced expression of steroidogenic markers such as *STAR* and *CYP17A1*, indicating increased androgen biosynthesis and contributing to the hyperandrogenic ovarian environment. Overall, single-cell transcriptomics revealed cell-type-specific gene expression changes involving hyperandrogenism and follicular dysfunction that contribute to PMOS pathogenesis [60].

A single-cell RNA sequencing transcriptomic study revealed modified TC subpopulations in PMOS, with enrichment of C2-STAR^high^ TCs and elevated expression of steroidogenic genes that promote androgen excess, including *STAR*,* CYP17A1*,* CYP11A1*, and *HSD3B*. The study identified dysregulation of the PI3K/AKT–LONP1–STAR axis, where reduced AKT signalling and decreased *LONP1* expression were associated with mitochondrial dysfunction, increased *STAR* activity, and hyperandrogenism. Additionally, the activation of oxidative stress response genes such as *ATF4* and *GSR* suggested altered redox regulation and mitochondrial stress in PMOS ovarian cells. These transcriptome results demonstrate how mitochondrial stress responses and TC metabolic reprogramming contribute to hyperandrogenism in PMOS [61].

Although the number of single-cell omics studies in PMOS remains limited compared with that of bulk omics approaches, these studies have provided unprecedented insights into cell-specific molecular alterations. By overcoming the averaging effect of bulk analysis, single-cell approaches have revealed distinct ovarian cell populations and regulatory programs involved in steroidogenesis, mitochondrial dysfunction, and follicular abnormalities, thereby improving our understanding of PMOS pathogenesis. (Refer to Table 4.)

**Table 4:** Summary of single-cell transcriptomics findings in PMOS

Single-cell type	Associated functional pathway	Key molecular changes	Contribution to PMOS pathogenesis	Reference
KRT8^high^ mural granulosa cells	Granulosa cell differentiation and folliculogenesis	Increased KRT8/KRT18 expression with enrichment of GC survival, differentiation, and follicular development signatures	Promotes abnormal follicular growth and hyperandrogenism	[60]
Theca interna and externa cells	Steroidogenesis and androgen biosynthesis	Elevated expression of STAR and CYP17A1	Enhances androgen production, contributing to ovarian hyperandrogenism	[60]
C2-STAR^high^ theca cells	Steroidogenesis–mitochondrial regulation axis	Increased STAR expression; dysregulation of PI3K/AKT–LONP1–STAR signalling	Drives androgen excess through increased steroidogenic capacity	[61]

## Discussion and conclusion

This review highlights how the integration of bulk and single-cell multimics provides advanced insights into theca and granulosa cell dysfunction in PMOS. Genetic susceptibility loci, abnormal DNA methylation patterns, and dysregulated non-coding RNA networks all contribute to altered steroidogenesis, folliculogenesis, and insulin signalling, according to evidence from genomics and epigenomics research. Genes related to androgen production, follicular development, metabolic regulation, inflammation, and cellular survival are also widely dysregulated, according to the transcriptomic analysis. Proteomic and metabolomic studies support these conclusions by providing functional evidence of disturbed protein networks and metabolic pathways linked to oxidative stress, insulin resistance, mitochondrial dysfunction, altered energy metabolism, and ovarian steroidogenesis. The intricate molecular interactions that underlie PMOS pathophysiology are highlighted by the convergence of various omics layers in a number of interrelated biological processes, including the PI3K-AKT, MAPK, TGF-β, and steroidogenic signalling pathways.

From a translational standpoint, several molecular candidates found in various omics studies have the potential to be therapeutic targets or biomarkers. Because of their roles in ovarian steroidogenesis and androgen biosynthesis, *DENND1A.V2*,* CYP17A1*, and *LHCGR* are especially promising. Similarly, circulating miRNAs such as miR-93-5p, miR-320a, miR-141-3p, and miR-486-5p may serve as minimally invasive biomarkers due to their associations with steroidogenic pathways and insulin signalling. Proteins, including NAMPT and AZGP1, as well as metabolites like L-tyrosine, L-tryptophan, and niacinamide, may also be useful indicators of metabolic dysfunction in PMOS.

Single-cell omics approaches complement bulk multiomics by resolving cell-type-specific alterations in PMOS pathogenesis. In GC, KRT8^high^ mural GC populations show enhanced differentiation and survival signals, contributing to follicular persistence. In TC, C2-STAR^high^ TCs with increased *STAR*,* CYP17A1*,* CYP11A1*, and *HSD3B* expression drive excess androgen biosynthesis. Stromal cells show a shift from TCF21^high^ to STAR^high^ populations, indicating extracellular matrix remodelling and increased steroidogenic activity. Overall, single-cell analysis identifies granulosa, theca, and stromal cell-specific dysregulation underlying steroidogenesis, mitochondrial stress, and ovarian dysfunction in PMOS.

Crucially, multiomics integration revealed that PMOS is caused by coordinated disruptions at several regulatory levels rather than isolated molecular changes. Multiomics techniques offer a more thorough framework for understanding disease mechanisms and discovering potential biomarkers and therapeutic targets by connecting genetic variation to downstream molecular and metabolic effects. However, functional studies to determine their mechanistic relevance must come after validation of these candidates’ clinical value in sizable, independent cohorts.

## Challenges, limitations and future perspective 

Despite significant advances in multiomics approaches, the translation of molecular discoveries in PMOS into a mechanistic understanding and clinical applications remains challenging. The majority of results from current proteomic, metabolomic, transcriptomic, and genomic research indicate relationships rather than causal processes, which is a significant drawback. Although several dysregulated genes, pathways, and metabolites have been identified, it is still unknown if these molecular changes are the fundamental causes of PMOS progression or secondary effects of the changed ovarian microenvironment [9]. Future research should use functional validation techniques, including animal models, ovarian organoids, CRISPR-based gene editing, and human GC and TC cell models, to determine causal links [62–64]. These methods assist in determining whether putative regulators directly impact important aspects of PMOS, including follicular arrest, oxidative stress, insulin resistance, hyperandrogenism, steroidogenesis, and mitochondrial dysfunction.

Another significant obstacle to interpreting multiomics results in PMOS is patient heterogeneity. PMOS is a highly heterogeneous disorder with diverse clinical phenotypes, including hyperandrogenic, obese, lean, insulin-resistant, and reproductive-dominant subgroups [65]. The generalisability of biomarkers and treatment targets may be limited if molecular changes found in one subgroup do not apply to the full PMOS population. Therefore, to identify the subtype-specific molecular signatures and enhance illness categorisation, future studies should involve larger, well-characterised cohorts with thorough clinical and molecular phenotyping. The incapacity of many existing methods to accurately represent the temporal and spatial complexity of ovarian dysfunction is another significant drawback.

While single-cell studies reveal cellular heterogeneity but frequently disturb tissue architecture and cell-cell interactions, bulk omics techniques provide averaged molecular profiles and may obscure cell-specific changes. A thorough understanding of how molecular changes develop during follicular development and how interactions between granulosa cells, theca cells, oocytes, immune cells, and stromal components contribute to PMOS pathogenesis will be possible through the integration of single-cell transcriptomics with spatial transcriptomics, imaging-based omics, and longitudinal sampling [66].

Molecular markers that show a consistent relationship with illness phenotype, functional significance, and accessibility in clinical samples should be prioritised for clinical translation. Biomarkers found in blood, extracellular vesicles, follicular fluid, or circulating metabolites may be useful for diagnosing PMOS, classifying phenotypes, predicting therapeutic response, and evaluating reproductive outcomes. Pathways involved in androgen biosynthesis, insulin signalling, mitochondrial dysfunction, oxidative stress, inflammation, and GC/TC regulation represent promising therapeutic targets. Future PMOS research should therefore move beyond descriptive molecular profiling toward integrated mechanistic models combining multiomics datasets, functional experiments, computational modelling, and clinical information. Such approaches will allow the identification of true disease-driving mechanisms, distinguish causal pathways from secondary changes, and facilitate the development of validated biomarkers and targeted interventions for ovarian dysfunction.

## Data Availability

No datasets were generated or analysed during the current study.

## Abbreviations

AR: Androgen Receptor
CYP17A1: Cytochrome P450 Family 17 Subfamily A Member 1
CYP19A1 (Aromatase): Cytochrome P450 Family 19 Subfamily A Member 1
DENND1A: DENN Domain Containing 1 A
DENND1A.V2: DENN Domain Containing 1 A Varient 2
DNMT1: DNA Methyltransferase 1
DNMT3A: DNA Methyltransferase 3A
DNMT3B: DNA Methyltransferase 3B
F2R: Coagulation Factor 2 Thrombin Receptor
FOXO: Forkhead box O
FSH: Follicle Stimulating Hormone
FSHR: Follicle Stimulating Hormone Receptor
GATA6: GATA-binding protein 6
IL12B: Interleukin-12 subunit beta
LH: Luteinizing Hormone
LHCGR: Luteinizing Hormone/Choriogonadotropin Receptor
PDCD4: Programmed Cell Death 4
PI3K–AKT: Phosphatidylinositol 3-Kinase -Protein Kinase B
RAB5B: RAB5B, Member RAS Oncogene Family
RBP4: Retinol Binding Protein 4
SPP1: Secreted Phosphoprotein 1
StAR: Steroidogenic Acute Regulatory protein
T: Testosterone
ZNF217: Zinc Finger protein 217
